# Repeat liver resection for pure large cell neuroendocrine carcinoma of the gallbladder: a favorable outcome

**DOI:** 10.1186/s12957-019-1666-9

**Published:** 2019-07-20

**Authors:** Ahmad Abutaka, Moamena El-Matbouly, Irfan Helmy, Walid Elmoghazy, Ibnouf Sulieman, Mohamed Ben Gashir, Madiha Soofi, Hatem Khalaf, Ahmed Elaffandi

**Affiliations:** 1 Department of General Surgery, Hamad General Hospital, Doha, Qatar; 2 Department of HPB Surgery and Liver Transplantation, Hamad General Hospital, Doha, Qatar; 3 Weill Cornell Medical College Qatar, Qatar Foundation, Doha, Qatar; 4 Department of Laboratory Medicine and Pathology, Hamad General Hospital, Doha, Qatar; 5 Department of Surgical Oncology, National Cancer Institute, Cairo, Egypt; 6 Department of Surgery, Sohag University, Cairo, Egypt

**Keywords:** Neuroendocrine tumors, Gallbladder cancer, Redo liver resection, Cholecystectomy, Large cell neuroendocrine cancer

## Abstract

**Background:**

The pure large cell type is a rare variant of primary neuroendocrine carcinoma of the gallbladder. Few reports have mentioned extended survival. Although a multimodal treatment has been described in the treatment of such rare disease, redo liver resection has not yet been mentioned.

**Case report:**

A 67-year-old lady was found to have poorly differentiated, high grade, pure large cell neuroendocrine tumor of the gallbladder after cholecystectomy for gallstones. After the diagnosis, staging workup showed a lesion in segment IVB/V of the liver, and chromogranin was elevated (982 mcg/L). The patient underwent central inferior hepatectomy and wedge excision of a lesion in segment III (discovered intra-operatively), with hilar lymphadenectomy. Three months after the first liver resection, she developed a new liver lesion II/III and underwent left lateral liver resection. The patient remained disease-free for 4 months following the second liver resection but then developed recurrent liver disease and was started on chemotherapy. Further progression led to multi-organ failure and death at 26 months from initial diagnosis.

**Conclusion:**

This is the first reported repeat liver resection in such a rare disease that has led to extended overall survival. We suggest that a group of selected patients with this rare malignancy, and liver-limited disease, may benefit from repeated liver resection.

## Introduction

LCNEC (large cell neuroendocrine tumor) of the gallbladder (GB) has rarely been reported in the literature [1]. The current WHO classification divides neuroendocrine neoplasms of the gallbladder into the categories of neuroendocrine tumor (NET G1 and G2), small cell neuroendocrine carcinoma (SCNEC), large cell neuroendocrine carcinoma (LCNEC), mixed adenoneuroendocrine carcinoma (MANEC), goblet cell carcinoid, and tubular carcinoid [2]. A limited number of reported cases has shown the dismal course of such disease that is even worse if surgery is not feasible [3]. With the lack of clear guidelines [4], the management of such patients is exceedingly challenging, especially in those with intrahepatic metastases; however, aggressive surgical approaches in selected patients have shown favorable outcomes [3]. Redo liver resections and their outcomes have hardly been mentioned in such a rare disease.

## Case report

**Fig. 1:**
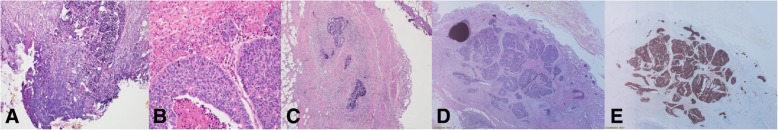
**a**–**c** Histopathology post-first liver resection showing features typical for neuroendocrine tumor. **d**–**e** The tumor cells that express chromogranin A

A 67-year-old lady with no prior comorbidities presented to Hamad General Hospital (HGH) with recurrent dull aching abdominal pain for 6 weeks with no history of weight loss, fever, or upper gastrointestinal (GI) symptoms. Initial abdominal imaging ultrasound (US) showed a distended gallbladder with a wall thickness of 5 mm, multiple stones (largest one measuring 16 mm), no intrahepatic duct dilatation, and no obvious liver lesions. She had an uneventful laparoscopic cholecystectomy with no significant intraoperative findings. The gallbladder was removed intact in an endobag, and there was no bile leak in the abdominal cavity. Histopathology of the gallbladder showed poorly differentiated (high grade) neuroendocrine carcinoma, large cell type, arising in a background of chronic and focally acute cholecystitis with cholelithiasis, intestinal metaplasia, and multifocal low-grade dysplasia. The tumor invaded through the muscular wall of the gallbladder into the surrounding adipose tissue with perineural and angiolymphatic invasion (Fig. 1a–c). As per new NCCN classification 2018, it is pT2bN0M0, stage IIb. Further immunohistochemistry staining showed that tumor cells were positive with pancyotkeatin (Ae1/Ae3), EMA, synaptophysin, chromogranin A, and CD56 but negative with CD15, WT1, TTF1, myogenin, and CD45 (Fig. 1d, e). These immunohistochemical stains confirm the epithelial lineage of this tumor and neuroendocrine differentiation.

**Fig. 2:**
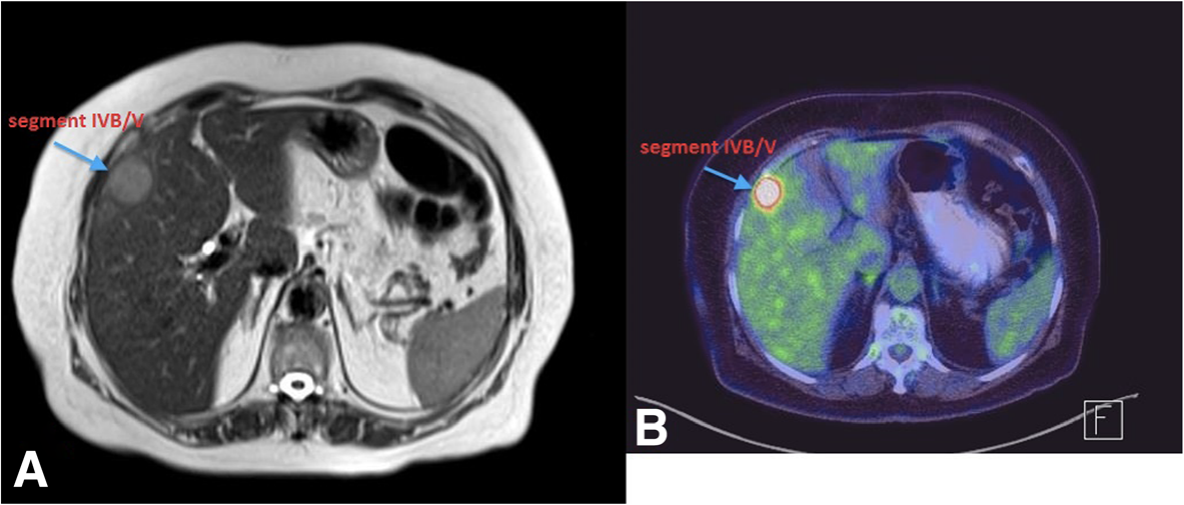
**a** MRI (prior to liver resection): a well-defined subcapsular hepatic focal lesion is noted at the right lobe segment IVB/V measures 28 × 27 × 30 mm, noted with capsule bulge with high T2 signal and low T1 signal. **b** PET CT (prior to liver resection): intensely hypermetabolic lesion in segment IVB/V of the liver consistent with malignancy just above the gallbladder fossa with slight contour bulge

**Fig. 3:**
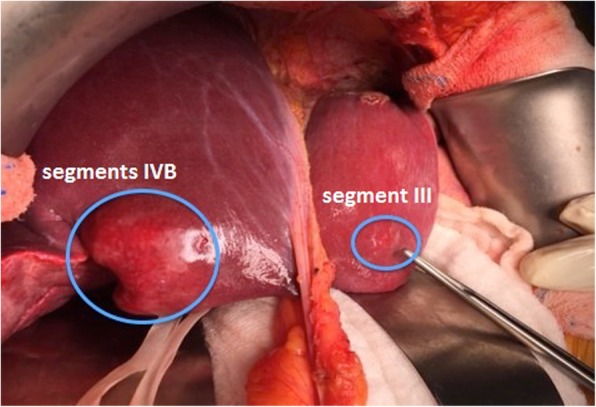
**a** Intra-operative findings of the lesion in segments IVB at the gallbladder bed with bulging at the bed of the gallbladder. **b** Segment III lesion that is incidentally discovered intra-operatively in the initial liver resection

Further imaging included a positron emission tomography scan (PET) that showed a hepatic lesion in segment IVB/V and magnetic resonance imaging (MRI) that also confirmed the existence of the same liver lesion in segment IVB/V measuring 28 × 27 × 30 mm (Fig. 2) with no evidence of disease elsewhere. Serum markers including chromogranin were found to be highly elevated (982 mcg/L). The case was discussed in the Hepato-Pancreato-Biliary (HPB) tumor board meeting and the decision was to carry out a completion hepatectomy and regional lymphadenectomy. Intra-operatively, a mass in segment IVB/V and another nodule in segment III were found (Fig. 3) that was not evident previously on PET CT or MRI. Intraoperative ultrasound examination of the liver confirmed the absence of other lesions; therefore, a central inferior hepatectomy was done together with wedge excision of the lesion in segment III and hilar lymphadenectomy.

**Fig. 4:**
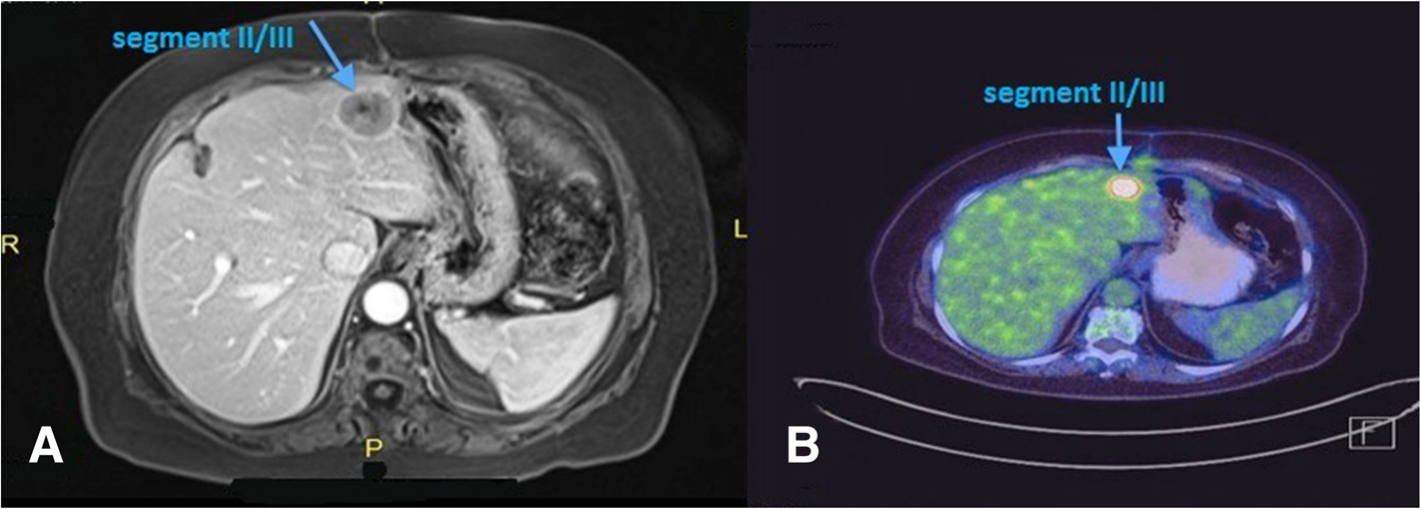
**a** MRI (4 months post-liver resection): focal lesion at segment II/III of the liver measuring 2.5 cm in diameter, with signal intensity and enhancement pattern impressive of metastasis. **b** PET scan 4 months post-liver resection: intense uptake in the left lateral aspect of the left liver lobe (segment II/III) corresponding to an approximately 2 cm sized hypodense lesion. New intense uptake corresponding to hypodense lesion in the left liver lobe consistent with new liver metastasis

Subsequent resection specimens showed similar features to those seen in the gallbladder confirming the metastatic disease (grade 3 neuroendocrine tumor—pure large-cell type) in the liver identical to the primary lesion within the gallbladder. The patient was kept under a rigorous follow-up schedule every 6 weeks. Four months following the first liver resection, a follow-up MRI showed a metastatic liver lesion 2.5 cm in diameter in segment II/III; therefore, a PET/CT scan was obtained to rule out any other metastatic foci, and none were found except the one detected by MRI in segment II (Fig. 4). Hence, a redo (second) liver resection was done (left lateral hepatic segmentectomy). Histopathology showed a single metastatic lesion of high-grade neuroendocrine tumor (pure large-cell type).

**Fig. 5:**
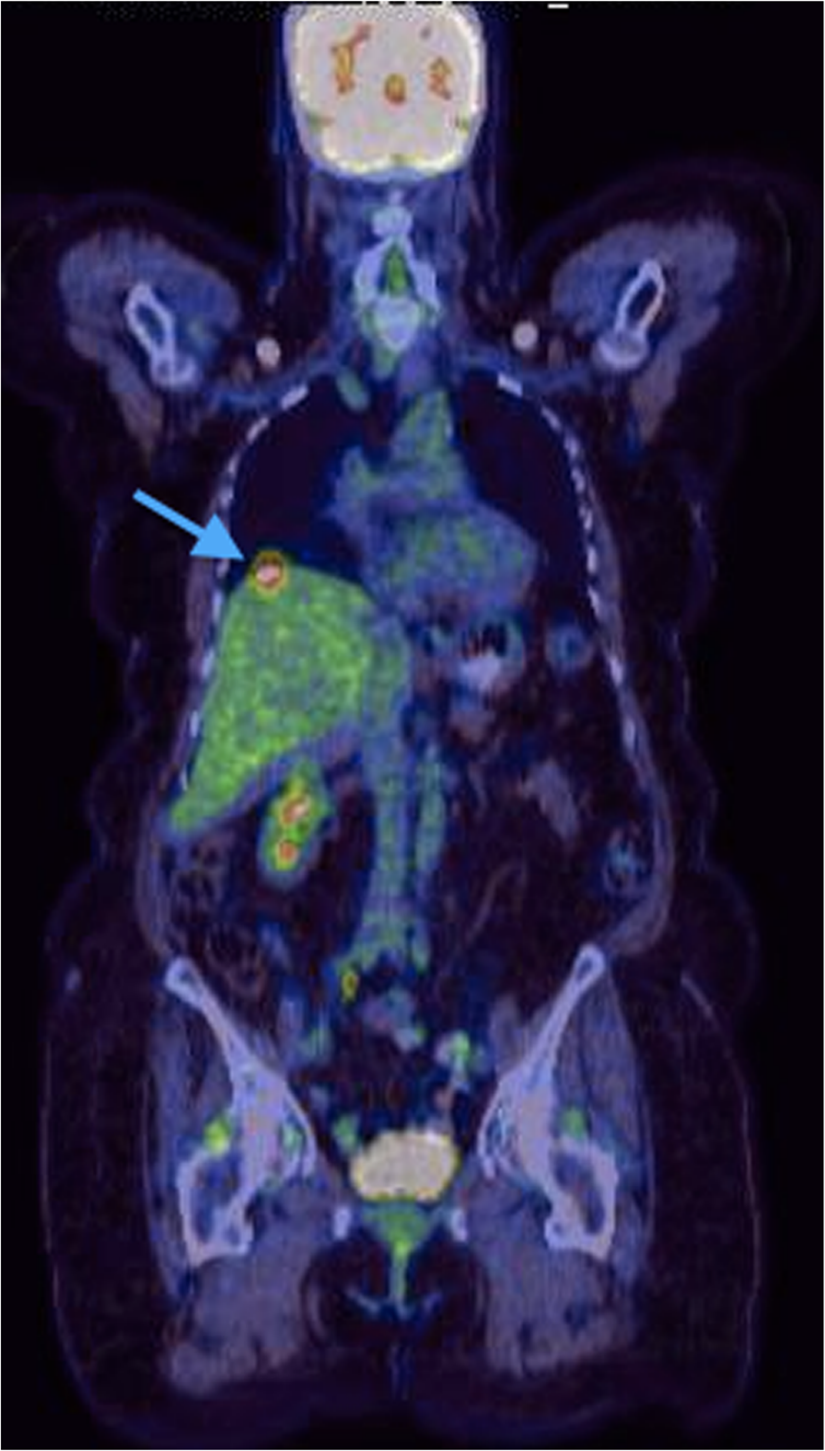
Follow-up PET CT showing evidence of recurrent liver disease localized to the liver (4 months post-redo liver resection): two focal liver uptakes (at the liver dome and in the surgical bed) consistent with recurrence

**Fig. 6:**
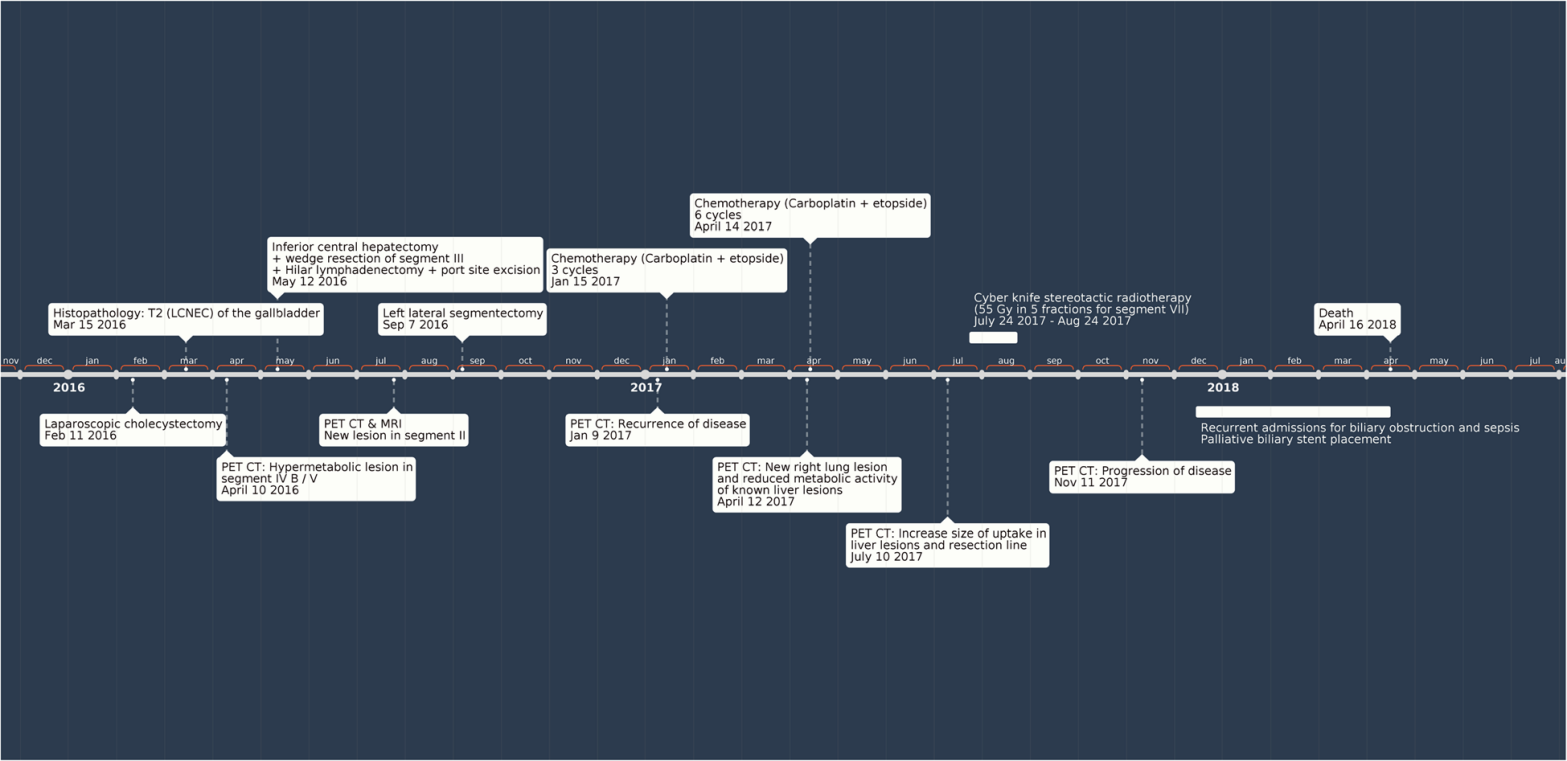
Timeline of the case investigations and management

Later during follow-up, the patient was found to have a locally recurrent disease on PET CT scan obtained 4 months post-redo (second) liver resection (Fig. 5). The patient received systemic chemotherapy regimen consisting of carboplatin and etoposide. After the third cycle, a follow-up PET CT scan showed a newly developed lung lesion with good response in the locally recurrent liver disease. Therefore, the patient received an additional 6 cycles of chemotherapy. A repeated PET CT scan showed progressive lung and liver disease. The patient started developing sepsis (cholangitis due to biliary obstruction); for which she had palliative biliary stent placement and succumbed 26 months after the initial diagnosis (Fig. 6).

## Discussion

Gallbladder cancer (GBCA) is known for its poor prognosis. A majority of the cases are diagnosed at an advanced stage. It is considered the most common malignancy of the biliary system and the fifth most common malignancy in the GI tract [5]. Primary neuroendocrine tumors (NETs) of the gallbladder present in a similar fashion to GBCA [3]. Primary neuroendocrine tumors (NETs) of the gallbladder are rare as they represent < 0.2% of all NETs [6]. Recently, the gastrointestinal and pancreaticobiliary neuroendocrine tumors have been classified on the basis of mitotic figures and Ki-67 index, regardless of the origin, size, or anatomic extent of the tumors [7, 8].

Pure large cell neuroendocrine carcinoma (LCNEC) has been first described in 2000 by M. Papotti et al. [9], who reported two cases of LCNEC, one of which was the pure type and the other is mixed with adenocarcinoma of the gallbladder. Around two-thirds of the histologically proven GBCA are incidentally found in cholecystectomy specimens [5, 10]. The determination of GB NETs is often difficult and requires many immunohistochemical expressions of markers as well as other cell type-specific amines and peptides. Our diagnosis of primary GB LCNEC has been based on the typical morphological and immunohistochemical features (Fig. 1). Normal gallbladder does not contain neuroendocrine cells, however, intestinal metaplasia in association with long-standing inflammation due to cholelithiasis and congenital anomalies may be the critical step in the development of NETs of the gallbladder [4].

In principle, complete surgical resection (R0) remains the gold standard for primary control of GBCA (T1/T2), the magnitude of which depends on the stage of the disease and may reach up to major liver resection. In the presented case, primary diagnosis of LCNEC was first reported post-cholecystectomy, which was confirmed later post-first liver resection. Completion surgery for LCNET is advocated in the absence of metastatic disease elsewhere. The spectrum of previously reported primary surgery for LCNEC started from simple cholecystectomy for T1a tumors and extended to hepatic resection and lymphadenectomy [4]. The presence of liver metastasis is a distinguishing feature of malignant NETs and is an important prognostic factor in patient survival [11]. Surgery has a primary role in the management of neuroendocrine liver metastasis (NELM) and its impact is well appreciated in both curative and palliative settings [12]. The role of chemotherapy and radiotherapy in the management of GB NETs and particularly those developing NELM is equivocal [3]; therefore, surgery remains the best treatment option if complete surgical resection (R0) of NELM can be achieved.

Pure LCNEC has been rarely mentioned (eight cases as of April 2016)^2^ and hence it becomes more challenging when deciding on the best management plan for LCNEC especially the surgical candidates as to whether concomitant adjuvant therapy is essential. Few reports have shown a limited survival benefit in those candidates receiving chemotherapy post-surgery [3] and for that reason, we have decided on the tumor board to advocate a close follow-up watchful policy post-first liver resection. Redo liver surgery, in general, has been extensively described in metastatic disease to the liver [13]. The influence of redo liver resection for patients having recurrent intrahepatic LCNEC remains unclear. However, a few case series on carcinoid and pancreatic NET, considered hepatic resection for neuroendocrine metastasis to the liver to be safe with observed extended survival and improved quality of life [14, 15]. We advocated repeated liver resection due to the absence of extrahepatic disease and good general condition of the patient along with the reported aggressive behavior of such neoplasms.

The longest reported survival of such cases was 69 months, as reported by Shimono et al. [16]. In their management approach, they adopted aggressive multimodal treatment options, which consisted of neoadjuvant intra-arterial chemotherapy and 3D radiotherapy followed by tri-segmentectomy and adjuvant chemotherapy. On follow-up, brain metastasis was managed by partial cerbellectomy and six gamma knife sessions. This report shows that aggressive approach may have contributed to improved survival.

Generally, the prognosis of NETs of the gallbladder depends on the grade of the tumor and the presence of hepatic metastasis. Most of the high-grade lesions are metastatic at diagnosis and only a few are resectable, and if we exclude the case reported by Shimono et al, the median survival is about 10 months [2]. Our patient survived for 26 months after the initial diagnosis. It is possible that other several factors may have contributed to the patient’s extended survival, such as a potential lead time bias or slightly different tumor grades.

## Conclusion

Redo liver resection has hardly been described in LCNEC of the gallbladder. This is the first reported repeat liver resection in such a rare disease that has led to extended overall survival. We can only speculate that a group of selected patients with such rare disease may benefit from a repeated liver surgery in the absence of extrahepatic disease.

## Data Availability

Data sharing not applicable to this article as no datasets were generated or analyzed during the current study.
